# Simple, Fast and Reliable Liquid Chromatographic and Spectrophotometric Methods for the Determination of Theophylline in Urine, Saliva and Plasma Samples

**Published:** 2014

**Authors:** Mohammad Charehsaz, Aylin Gürbay, Ahmet Aydin, Gönül Şahin

**Affiliations:** a *Yeditepe University, Faculty of Pharmacy, Department of Toxicology, Istanbul, Turkey.*; b *Hacettepe University, Faculty of Pharmacy, Department of Toxicology, Ankara, Turkey.*

**Keywords:** Theophylline, Plasma, Saliva, Urine, HPLC, UV spectrophotometry

## Abstract

In this study, a high-performance liquid chromatographic method (HPLC) and UV spectrophotometric method were developed, validated and applied for the determination of theophylline in biological fluids.

Liquid- liquid extraction is performed for isolation of the drug and elimination of plasma and saliva interferences. Urine samples were applied without any extraction. The chromatographic separation was achieved on a C18 column by using 60:40 methanol:water as mobile phase under isocratic conditions at a flow rate of 0.75 mL/min with UV detection at 280 nm in HPLC method. UV spectrophotometric analysis was performed at 275 nm.

The results of HPLC analysis were as follows: the limit of quantification: 1.1 µg/mL for urine, 1.9 µg/mL for saliva, 3.1 µg/mL for plasma; recovery: 94.85% for plasma, 100.45% for saliva, 101.39% for urine; intra-day precision: 0.22–2.33%, inter-day precision: 3.17-13.12%. Spectrophotometric analysis results were as follows: the limit of quantitation: 5.23 µg/mL for plasma, 8.7 µg/mL for urine; recovery: 98.27% for plasma, 95.25% for urine; intra-day precision: 2.37 – 3.00%, inter-day precision: 5.43-7.91%.

It can be concluded that this validated HPLC method is easy, precise, accurate, sensitive and selective for determination of theophylline in biological samples. Also spectrophotometric analysis can be used where it can be applicable.

## Introduction

**Figure 1:**
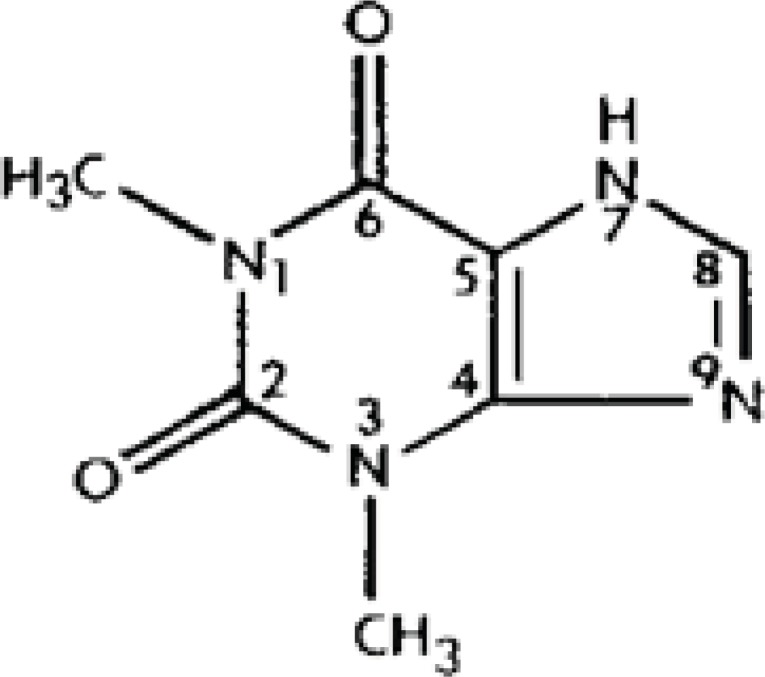
Chemical structure of theophylline

Theophylline, 1,3-dimethylxanthine, (Figure 1) has been widely used as a bronchodilator drug in the treatment of asthma and chronic obstructive pulmonary disease (COPD) due to its cheapness and effectiveness. 

There is a strong relationship between bronchodilator effect and blood levels of theophylline. Maintenance of its blood level between 10-20 µg/mL is important to obtain a maximum bronchodilator effect; its serious side effects take place over 20 µg/mL; its efficacy falls below 10 µg/mL of blood concentrations. Besides having a narrow therapeutic range, alteration of its pharmacokinetic characteristics due to individual variations, drugs, diet and habits such as smoking and alcohol has led to requirement of a simple, sensitive and reproducible assay method for determination of theophylline and its metabolites (1,2). Biological samples such as urine plasma, serum and tissue have been used in determination of theophylline for doping control investigations, clinical pharmacokinetic experiments and human liver metabolism studies (3).

Although in recent years, theophylline has been determined using a number of techniques including high-performance liquid chromatography (HPLC) (2-10), spectrophotometry (11-13), thin layer chromatography (TLC) (14), high-performance thin layer chromatography (HPTLC) (15), gas-liquid chromatography (GLC) (16), but some of them suffer from poor resolution, long chromatographic run time and tedious pretreatment of the samples, such as repeated extraction, evaporation and reconstitution procedures (17-21). The method proposed by Dockendorff *et al*. in 1998 using automated solid phase extraction system for theophylline can be thought a little bit time consuming. (18). Labacevski *et al*. proposed an extraction method which follows organic extraction, evaporation to dryness at 40 ^º^C under a gentle stream of nitrogen and reconstitution in methanol (7). Zhai *et al*. described a liquid chromatographic method for the simultaneous determination of theophylline, enoxacin and ciprofloxacin in plasma and saliva. The extraction procedure and mobile phase was a little bit complex and the flow rate was also high (22). Muir *et al*. developed a long and complicated method which requires an ion-pair gradient elution (23). Also the method proposed by Weinberger and Chidsey requires a constant temperature of 55 ^º^C to be maintained with a column oven during the run time (19).

We aimed to develop a simple and suitable analytical method for the determination of theophylline in biological fluids which can overcome the possible handicaps of above mentioned methods. 

## Experimental

*Chemicals*

Theophylline (1,3-dimethylxanthine) was provided as a gift by Nobel pharmaceutical company (Turkey). Caffeine, theobromine (3,7-dimethylxanthine), methanol and isopropanol were purchased from Sigma (Sigma Co Ltd., St. Louis, MO, USA). Chloroform and ammonium sulphate was obtained from Carlo Erba (Italy). Bis de-ionised water was used throughout analysis. Methanol was HPLC grade and all other solvents were of analytical-reagent grade.

*Sample Preparation*

Stock solution of theophylline (1 mg/mL) was prepared in the methanol and stored at -20 °C for 2 month and protected from light.

In order to investigate the effects of medium on calibration curve linearity and equation parameter, working standard solutions of thoephylline were prepared in methanol, water, urine, plasma and saliva matrices.

Human urine, saliva and plasma samples were obtained from healthy volunteers and stored at -20 °C until analysis. The subjects abstained from any xanthine-containing food or beverages or alcoholic products for 48 h prior to sample collection. Orange- flavoured lozenges served as a reflex stimulus to induce salivation.

For preparation of calibration curves, methanol, urine, saliva and plasma samples were diluted 1:10 with bis de-ionised water and were spiked with theophylline stock solution daily at final concentration of 1, 2, 5, 10, 20 and 50 µg theophylline per mililiter for HPLC analysis. In spectrophotometric analysis, calibration curve was studied at the final concentrations of 5, 10, 20, 50 and 100 µg/mL in 4 different spiked matrices (water, methanol, urine and plasma) daily.

Quality control (QC) samples were prepared at the concentrations of 3, 10, 15 and 30 µg theophylline per mililiter in the same matrices for both analysis methods. 

*Determination of* *Theophylline by HPLC*

*Extraction*

Spiked urine samples were mixed on a vortex mixer for 30 seconds at maximum velocity and centrifuged at 1400 rpm for 2 min. Then supernatant was filtered through a 0.2 µm membrane filter and was used for analysis.

Spiked plasma and saliva samples were extracted according to method described by Jonkman *et al*. method (10). Briefly 0.5 mL plasma or 1.0 mL saliva (because saliva concentrations are about 50% of plasma concentrations) was placed into 10 mL screw capped centrifuge tube. 0.3 mL of a saturated ammonium sulphate solution was added and mixed on a vortex mixer for 10 seconds at maximum velocity. Then 2.0 mL of a chloroform-isopropanol (20:1, v/v) mixture was added, mixed in a vortex for 1 minute, centrifuged at 3500 rpm for 5 minutes and the aqueous layer was aspirated. The organic layer was filtered through a 0.2 µm membrane filter and was used for analysis.

*Equipment*

The analytical HPLC system employed was an Agilent 1100 Series HPLC equipped with an Agilent 1100 Series diode-array detector (Agilent Technologies, Germany). The HPLC pumps, autosampler and diode-array system were monitored and controled using the HP Chem Station computer program (Agilent). A reversed-phase analytical column (ODS 2-Spherisorb C18, 25 cm x 4.6 mm, 5µm) was used.

*Mobile phase*

The mobile phase, containing methanol and bis de-ionised water (60:40, v/v), was prepared and degassed by vacuum prior to use. Separation was done at room temperature. Guard column cartridge (4.3x10mm ODS-C18, 5 µm particle size) was placed just before the analytical column to reduce contamination. The isocratic separation was performed at 0.75 mL/min flow-rate.

*Injection volume, run time and detection*

Injection volume was 20 µL with run time of 5 minutes and eluted peaks were detected at 280 nm. Peak-height was used to determine theophylline concentration in samples.

*Determination of* *theophylline by s* *pectrophotometry*

*Extraction*

For spectrophotometric analysis, 100 µL of spiked plasma or urine samples were transferred to a glass tube and 300 µL of water was added. Then 8 mL extraction solvent (chloroform/isopropanol, 20:1, v/v) was added and mixed in a vortex for 30 seconds. After centrifugation (3500 rpm for 5 minutes) and discarding upper aqueous phase, 6 mL of the organic layer was transferred to a clean tube. Then 1 mL of 0.3 mol/L NaOH was added and mixed in a vortex for 30 seconds. After centrifugation (3500 rpm for 5 minutes), 0.8 mL of the aqueous phase was transferred to a 1.0 mL quartz cuvette and 40 µL of 2 mol/L NH_4_Cl solution was added and mixed. The absorbance at 275 nm against blank was recorded (24).

*Equipment*

Spectrophotometric measurements were made on Shimadzu model UV 1601 double beam UV Visible spectrophotometer with matched quartz cuvettes.

*Analysis of validation parameters*

*Assay precision and accuracy*

The precision and accuracy of both methods were evaluated by performing replicate analyses of QC samples (3, 15 and 30 µg/mL for HPLC; 10, 15 and 30 µg/mL for spectrophotometry) in biological fluids. The precision for each matrix was calculated as the relative standard deviation (RSD%) of inter-day and intra-day measurements. For intra-day precision, all measurements were repeated five different times in the same day. For inter-day precision, all measurements were replicated in 7 consequent days for each matrices. Then RSD% values were calculated for intra-day and inter-day measurements. The accuracy of the method were evaluated by recovery percentage of spiked samples.

*Limit of detection (LOD) and limit of quantification (LOQ)*

LOD and LOQ of both methods were determined from the calibration curve, using the following equation: 3б/m and 10б/m, where б is the standard deviation of intercept (*b*) and *m* is the slope of the calibration curve.

*Selectivity*

Selectivity of the method was evaluated in human urine and saliva samples spiked with mixed standard solutions containing theophylline, caffeine and theobromine.

*Effect of urine pH-values on theophylline measurement*

As the pH of urine can normally vary between 4.5 and 8, the influences of pH changes on theophylline measurement were investigated. 

Urine samples with pH values of 4.5, 6 and 8 were prepared and spiked with theophylline stock solution in final concentrations of 15, 60 and 150 µg/mL. The absorbances of three different concentrations of these solutions in three different pH were compared by spectrophotometric analysis.

*Statistical analysis*

The statistical comparisons were done by SPSS (Statistical Package for the Social Sciences) computer program. The comparisons of the calibration curve slopes were performed by Student’s t-test. The effect of urine’s pH changes on the measurements was evaluated by one way ANOVA test. 

## Results

*Retention Times and Chromatograms*

**Figure 2:**
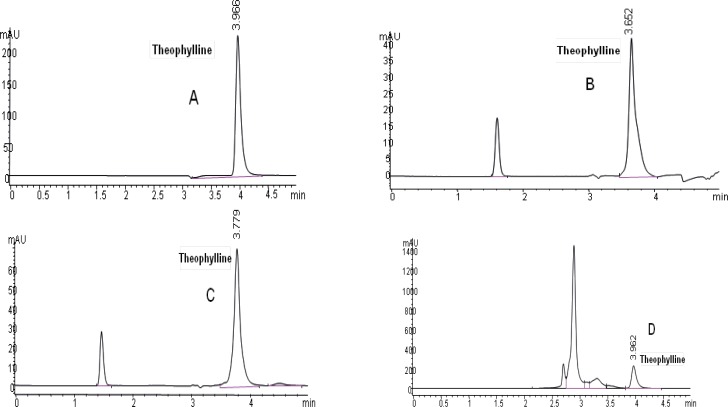
Chromatograms of theophylline (20 µg/mL): (A) in methanol solution, (B) in plasma sample, (C) in saliva sample and (D) in urine sample

Theophylline chromatograms obtained by HPLC in 4 different matrices were shown in Figure 2.

Observed mean retention times (mean ± SD, n=50) were 3.98 ± 0.041, 3.58 ± 0.098, 3.84 ± 0.52, 3.97 ± 0.027 in methanol, plasma, saliva and urine containing theophylline in five consequent days, respectively. 

*Linearity*

Calibration curve equations for HPLC analysis were shown in Table 1. According to the correlation coefficient (r^2^) values, calibration curves were linear. 

**Table 1:** The calibration curve equations and r^2^ values in HPLC analysis

**Matrix **	**Calibration curve equations (n=10)**	** r** ^2^
Methanol*	y=10.9231 (±0.5132)x – 2.2728 (±0.0013)	0.9998 (±0.0004)
Plasma	y=2.4218 (±0.0108)x – 1.1622 (±0.7465)	0.9985 (±0.0004)
Saliva	y=4.2294 (±0.2566)x – 1.8549 (±0.7976)	0.9996 (±0.0004)
Urine	y=11.1550 (±0.2723)x – 5.0195 (±1.2026)	0.9998 (±0.0002)

n: the number of calibration curve replication. values were represented as y=mean slope(±s.d.)x – mean intercept (±s.d.).

*: p<0.05 when compared with plasma and saliva.

In spectrophotometric analysis, a good linearity was also observed in calibration curves for four different matrices. Standard calibration curve equations and correlation coefficient (r^2^) values were shown in Table 2.

**Table 2:** The calibration curve equations and r^2^ values in spectrophotometric analysis

**Matrix **	**Calibration curve equations (n=10)**	** r** ^2^
Methanol*	y=0.0036 (±0.0002)x – 0.0130 (±0.007)	0.9994 (±0.0005)
Water	y=0.0042 (±0.0002)x – 0.0061 (±0.0050)	0.9992 (±0.0024)
Plasma	y=0.0034 (±0.0002)x – 0.0053 (±0.0032)	0.9993 (±0.0005)
Urine	y=0.0039 (±0.0003)x – 0.0113 (±0.0102)	0.9991 (±0.0081)

n: The number of calibration curve replication. Values were represented as y=mean slope(±S.D.)x – mean intercept (±S.D.).

* :p<0.05 when compared with plasma.

When the equations of calibration curves obtained by HPLC was compared, the slope of calibration curve for urine matrix was not found different (p > 0.05) from that of methanol. On the contrary, the slope of calibration curve in methanol was significantly different from saliva and plasma matrices (p < 0.05). With these results, calibration solutions can be prepared in methanol for theophylline analysis in urine, but in case of theophylline analysis in saliva and plasma, calibration solution matrices should be compatible with sample matrices. These results were similar with that of spectrophotometric analysis.

*Precision*

The precision for each matrix was given by the percentage of inter-day and intra-day RSD% values and were summarized in Table 3a and 3b.

**Table 3a:** RSD% values of intra-day and inter-day measurements as precison of the assay for HPLC method

**QCSample Concentration (µg/mL)**	**In Urine**		**In Saliva**		**In Plasma**	
	**inter-day (n=7)** *	**intra-day (n=5)** **	**inter-day (n=7)** *	**intra-day (n=5)**	**inter-day (n=7)** *	**intra-day (n=5)**
3	13.12	0.23	4.02	2.33	4.15	0.65
15	4.08	0.25	6.86	0.45	8.71	1.68
30	5.93	0.22	5.39	1.42	3.17	2.14
Mean±SD	7.71 ± 4.77	0.23 ± 0.015	5.42 ± 1.4	1.4 ± 0.94	5.34 ± 2.96	1.49 ± 0.76

* : number of days;

** : number of replications.

**Table 3b:** RSD% values of intra-day and inter-day measurements as precison of the assay for spectrophotometry

**Qc Sample Concentration (µg/mL)**	**In Urine**		**In Plasma**	
	**inter-day** **(n=7)** *	**intra-day** **(n=5)** **	**inter-day** **(n=7)**	**intra-day** **(n=5)**
10	11.57	4.25	6.3	4.14
15	5.68	2.29	4.03	1.29
30	6.48	2.47	5.95	1.68
Mean±SD	7.91 ± 3.19	3 ± 1.08	5.43 ± 1.22	2.37 ± 1.54

* : number of days;

** : number of replications.

**Table 4:** Accuracy of HPLC analysis as recovery percentage

**Urine theophylline** **concentration (µg/mL)**				**Saliva theophylline concentration (µg/mL)**				**Plasma theophylline concentration (µg/mL)**			
**IC** *	**Add**	**Found±SD (n=5)**	**R%** **	**IC**	**Add**	**Found±SD (n=5)**	**R%**	**IC**	**Add**	**Found±SD (n=5)**	**R%**
0	3	3.2 ± 0.3	106.7	0	3	3.22 ± 0.2	107.3	0	3	2.82 ± 0.24	94
0	15	14.76 ± 0.22	98.4	0	15	14.37 ± 0.58	95.8	0	15	14.51 ± 0.62	96.73
0	30	29.72 ± 1.53	99.07	0	30	29.47 ± 2.16	98.23	0	30	28.15 ± 1.87	93.83
**Mean R%±SD: 101.39 ± 4.6**				**Mean R%±SD: 100.45 ± 6.08**				**Mean R%±SD: 94.85 ± 1.63**			

* IC: initial concentration,

** R:recovery.

Mean accuracy values of applied spectrophotometric method for plasma and urine samples at concentrations of 10, 15 and 30 µg/mL were 98.27% ± 3.71 and 95.25% ± 9.27 (mean R% ± SD, n=5) respectively.

*LOD and LOQ*

In chromatographic analysis, LOD was found 0.3, 0.6 and 0.9 µg/mL while LOQ was found 1.1, 1.9, 3.1 µg/mL for urine, saliva and plasma respectively.

In spectrophotometric analysis, the LOD was found 2.61 and 1.57 µg/mL while the LOQ was found 8.7 and 5.23 µg/mL for urine and plasma respectively.

*Selectivity*

**Figure 3:**
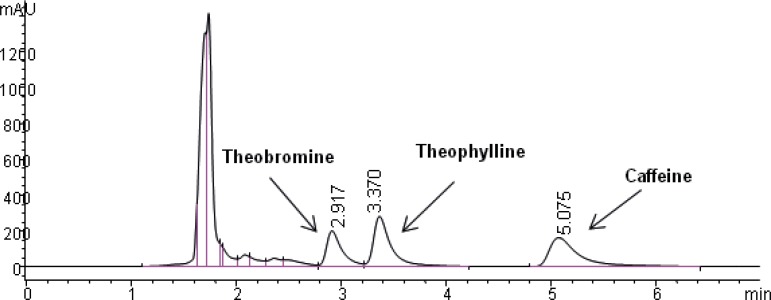
Chromatogram obtained with blank urine which was spiked with theobromine (30 µg/mL), theophylline (50 µg/mL) and caffeine (50 µg/mL).

**Figure 4:**
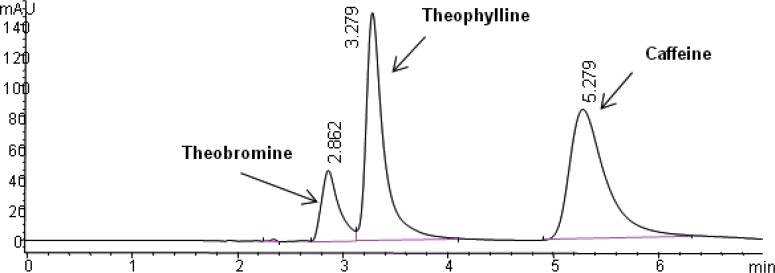
Chromatogram obtained with blank saliva which was spiked with theobromine (30 µg/mL), theophylline (50 µg/mL) and caffeine (50 µg/mL).

The selectivity of the method was found to be satisfactory. No disturbing peaks were found in urine and saliva samples (n=30). The separation of theophylline from caffeine and theobromine (as shown in Figure 3 and 4) was succeeded. Since theobromine and caffeine have different retention times (~2.5 and 5 minutes respectively), they do not interfere with theophylline in this procedure ( the resolution between theophylline and theobromine was greater than 1.5).

*Effect of urine pH-value on theophylline measurement*

The influences of pH changes of urine samples on theophylline measurement were investigated in three different pH and no significant effects were observed.

## Discussion and Conclusion

As other xanthines such as caffein, theobromine and their metabolites have the same chromatographic properties with theophylline, a system with high separation capacity is required for a selective theophylline assay. Comparing the methods described by Labacevski *et al.* (7) and Dockendorff *et al.* (18), in the present study, extraction method described by Jonkman *et al*., a simple one-step extraction procedure with chloroform-isopropanol without further clean up or preconcentration, was used for plasma and saliva (10). Also, urine samples were applied without any extraction, and column clogging or deterioration did not occur during all analysis. Some methods are described which involve direct injection of deproteinised serum (25-27). This application has the disadvantage of column deterioration.

The simplicity of elution procedure, use of an economical and readily available mobile phase (methanol: water), UV detection (280 nm), and use of the same mobile phase for three different biological fluids, in addition to absence of internal standard are another advantages of the current technique comparing to another methods (22,23). 

The short run time in this method allows us to do theophylline analysis relatively in a short time. Tajerzadeh *et al.* described a selective assay which separates theophylline and its major metabolites, but this method has a longer retention time (~8 min) than that of the current method (~4 min) (2).

Also in contrast to Weinberger and Chidsey method (19), analyses were performed at the room temperature in this method. 

When we looked at the pharmacopeia monographs for theophylline assay, this proposed method have the similarities to the United States Pharmacopeia (USP) assay method, such as flow rate, injection volume and detection at 280 nm. But the mobile phase in this method is more convenience than the mobile phase of USP method (acetonitrile in buffer solution consist of sodium acetate trihydrate and glacial acetic acid) (28). European Pharmacopeia (EP) does not have such kind of analysis method (29). 

Validation parameters with high accuracy (94-101% recovery), acceptable reproducibility with a good precision (6.16±3.12% interday and 1.04±0.86% intraday RSD%) and high sensitivity (LOQ: 1.1 µg/mL for urine, 1.9 µg/mL for saliva and 3.1 µg/mL for plasma) were obtained with the present HPLC analysis. Although the inter-day reproducibility was found a little bit high at the lowest concentration for urine, but this value is also very near to the acceptable RSD value (1%-10%). 

Validation parameters of the spectrophotometric method were as follows: mean values of accuracy for plasma and urine samples (95-98% recovery) and precision (6.67±2.55% interday and 2.69±1.24% intraday RSD%). However, LOQ was 5.23 µg/mL for plasma and 8.7 µg/mL for urine. When this LOQ value compared with HPLC analysis, it was found slightly higher. With these results spectrophotometric analysis of theophylline in biological matrices can be used, when HPLC analysis conditions are not available.

We also compared the equations of calibration curves for four different matrices (Table 1 and 2). According to the present results, it could be concluded that it is not necessary to prepare standard solutions in urine matrix in place of methanol or water for urine theophylline analysis by HPLC and spectrophotometry. In case of saliva and plasma analysis, calibration solutions should be prepared in the same sample matrices.

Since different pH values (4.5, 6 and 8) had no effect on theophylline measurement by spectrophotometric method, it could be concluded that the present method is suitable for theophylline analysis in urine samples. The reason is that acid dissociation constant value of theophylline is 8.70 (30). In all studied pH values which are in the range of urine pH, solutions are predominated by the protonated form of the theophylline. Therefore, the same species is determined and no change is observed in spectrophotometric measurements.

In addition, saliva theophylline analysis can be applied easily in order to monitor the drug levels especially in pediatric patients since it is not an invasive sampling method and contain 50% of serum drug level. 

As a conclusion, this proposed HPLC method can be used for quantification of theophylline in urine, blood and saliva as an efficient, selective, rapid and simple method. Also the spectrophotometric analysis can be used in clinical laboratories where an efficient HPLC system is not available. 
